# Supplementary material for: Learning curve and surgical outcome of robotic assisted colorectal surgery with ERAS program
Source: Sci Rep. 2022 Nov 29;12:20566. doi: 10.1038/s41598-022-24665-w (PMC9709162; doi:10.1038/s41598-022-24665-w)
Supplement: Supplementary file 2 — Supplementary Information 2. [file 41598_2022_24665_MOESM2_ESM.zip › RAL Raw data-Ñ[▒K/057/OP_ñw╝╨░O▒Kñσ.pdf]

正式摘要(電子簽章：000000210046933)
請選擇...reporter

臺中榮民總醫院

Operation Note

[redacted]

- 手術日期：2021/01/27
- 手術醫師：林俊余，CR 林陳彥，R 林辰祐
- Anesthesia : GE
- Position : Lithotomy
- Aseptic agents : Betadine Aqua , Zephnan

Diagnosis

- Pre-operative diagnosis : Malignant tumor of D-colon
- Post-operative diagnosis : s/p robotic left hemicolectomy

Surgical Indication

- For removing the primary tumor

Operation

- 1. Robotic (XI) assisted left hemicolectomy
  2. Intraoperative colonoscopy : (-)
  3. High ligation (+) : At IMA root. Left colic artery and IMV were also ligated
  4. Take down from the splenic flexure : (+)
  5. Robotic endoGIA (45-4.3) X2 + EEA (29mm) were applied
  6. Proximal cut end: 6 cm, distal cut end: 6 cm
  7. Indocyanine green agent was administered for testing during operation.
  8. No drainage tube was inserted
  9. Hyalobarrier gel was applied below the extended wound over the LLQ of the abdomen

Finding

- 1. An ulcerative, about 4 X 4 cm tumor was located at distal D-colon
  2. Mild adhesion was noted between the D-S colon and the nearby abdominal wall
  3. No obvious ascites was noted
  4. Several serosa tear sites were noted over the T-D junction while performing take-down procedure of the splenic flexure. It was all reparied with interrupted sutures via 3-O mersilk.
  5. For better visualization, another assisted port was created over the LLQ of the abdomen. It was later further extended to take out the specimen.
  6. Indocyanine green test result :
  A. Prior to the resection of S-colon (Distal of the specimen) : About 36 seconds
  B. Prior to resection of proximal D-colon (Proximal of the specimen) : About 25 seconds
  C. After the anastomosis was nearly completed (Right before firing EEA) : About 33 seconds
  7. Bilateral doughnuts : intact ; Air leak test: Done and no air leak was found.

Procedure

- 1. General endotracheal anesthesia was induced and then the patient was placed in modified Trendelenburg lithotomy position.
  2. Preoperative antibiotics were given. A Foley catheter and nasogastric tube were placed.
  3. The abdomen was prepped and draped in standard sterile fashion.
  4. A transverse incision was made over the right side of the umbilicus and a 12 mm trocar was

- inserted into the peritoneal cavity by open method. Peumoperitoneum was established with CO2 inflation.
  5. Laparoscopy was inserted and then other two 8mm trocars were inserted over LUQ area of abdomen, also another one over RLQ area of abdomen. Finally, a 12mm trocar was inserted over RUQ area as an assist-port. The operative findings were mentioned as above. After the setting was completed, the laparoscopy was shifted to the LUQ 8mm trocar site instead.
  6. The sigmoid colon was held by grasper and the peritoneum overlying the mesentery was scored with electrocautery. The adhesion bands were resected carefully.
  7. The inferior mesentery artery was identified, ligated above the left colic artery with endoclips, and transected.
  8. Dissection was done above the Gerota's fascia and toward the peritoneal attachments.
  9. The colon was mobilized from retroperitoneum overlying the sacral promontory and into the pelvis.

  10. Peritoneal reflection was not resected.
  11. Distal descending colon and s-colon were freely mobilized after the mesoclon was all dissected.
  12. The avascular line of Toldt along the left paracolic gutter was incised from the sigmoid colon to splenic flexure as possible. The instrument (suction tube) could not reach the splenic flexure site from the original assistant port position.
  13. For adequate visualization of the operaive field, another 5mm trochar was inserted made over the LLQ of the abdomen, to enbale better drainage and traction function.
  14. Finally, the splenic flexure was taken down.
  15. Indocyanine green agent was administered from IV line. The blood supply of the colon was examed carefully.
  16. The proximal s-colon was divided with robotic endoGIA (45-4.3) X2.
  17. The desired proximal resection site was selected over the proximal D-colon area and marked via electrical cautery. Indocyanine green agent was then administered again from IV line, to make sure it has an excellent blood supply
  18. A new incision was made over LLQ area of abdomen (Extended from the LLQ assistant port area.). We approached into the peritoneal cavity by open method. D-S colon was pulled out.
  19. The proximal colon was then taken out for carefully inspection. The mesenteric fat was gently cleared from the terminal 2-3 mm of the bowel ends.
  20. The field was isolated with moist pad and the bowel opened and sized. It was determined that a 29 mm circular stapler could be accommodated. A purse string suture of 2-0 prolene was placed. The tumor-containing tissue was removed. Then the anvil was inserted, followed by the purse string suture. The colon was then put back to the abdominal cavity.
  21. Pneumoperitoneum was constructed again.
  22. The anal canal was irrigated with distil water and then the circular stapler was introduced through the anus and pierced through the upper area (The ventral site of the body) of the stapler line.
  23. The proximal bowel with anvil in place was engaged with the circular stapler. Indocyanine green agent was administered for the last time from IV line to check the bloody supply status. Then, the stapler was fired. Two complete donuts were obtained and the distal one was submitted to pathology for margin confirmation.
  24. The anastomosis was tested for leaks with irrigation solution and air introduced through the rectum and was found to be patent and intact.
  25. Bleeder was checked thoroughly again and the blood clot was drained out as possible.
  26. Hyalobarrier gel was applied below the extended wound over the LLQ of the abdomen. The fascia was then closed with interrupted sutures via Num.1 Vicryl.
  27. The fascia of the remaining trochar wounds were all closed with interrupted sutures via 1-O Vicryl.
  28. Massive N/S was applied to irrigate the wound.
  29. The skin wound was sutured with interrupted method via 3-O Nylon, then covered with Medipore.

  30. The patient tolerated the procedure well and was brought to the POR in stable condition.
- Estimated blood loss: 100 c.c
- 器械數量: 正確
- 紗布數量: 正確

Image

- PAINT
  - abdomen

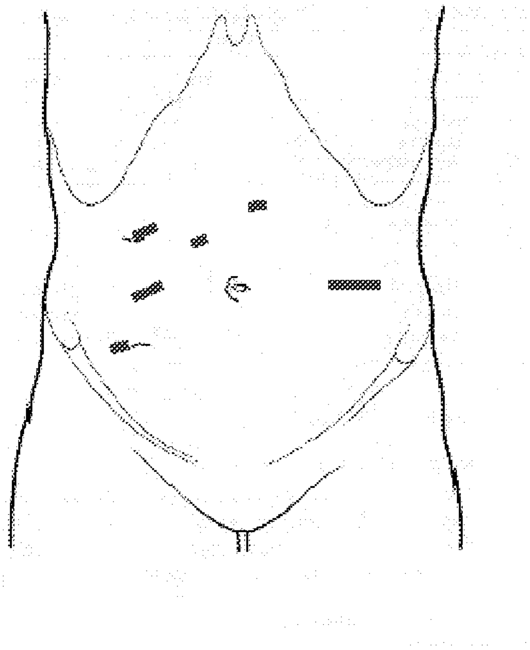

- colon & rectum

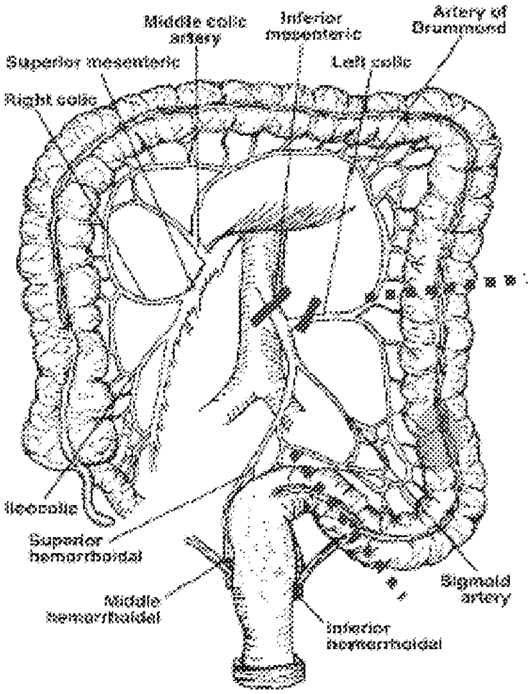

- The resected rectum

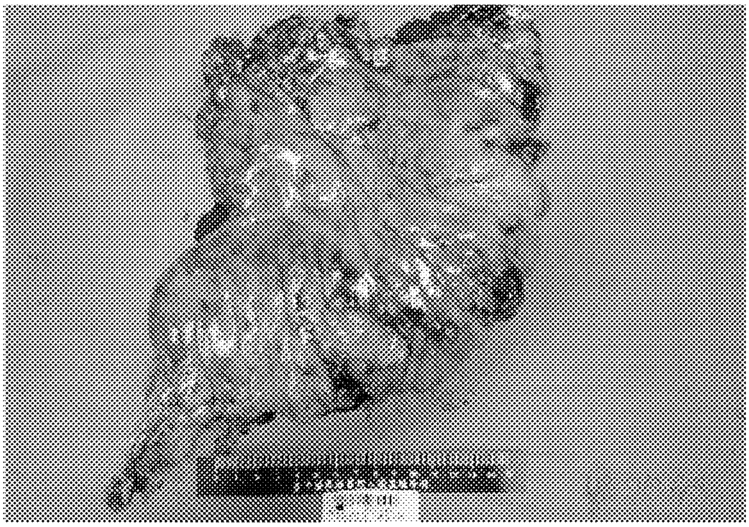

The tumor could be clearly seen

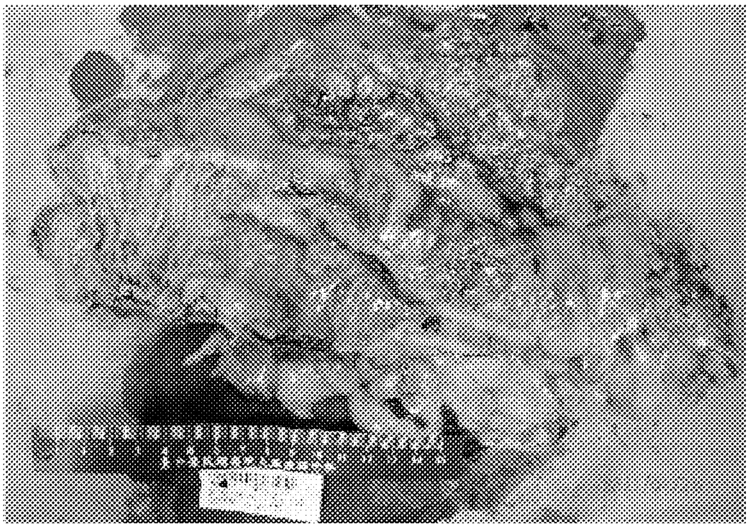

主治 2222H林俊余 / 醫師 2301H林陳彥

正式摘要(電子簽章：000000210046933)

請選擇...reporter

doctorRank:B
